# Decreased serum MMP-9 levels in patients with nontraumatic osteonecrosis of the femoral head

**DOI:** 10.1186/s12891-023-06342-9

**Published:** 2023-03-29

**Authors:** Guopeng Li, Fengxuan Ji, Wenchao Guo, Biaofang Wei

**Affiliations:** 1 Weifang Medical University, Weifang, Shandong Province China; 2 Department of Orthopedics, Linyi People’s Hospital, Jie Fang Road East, No.27, Linyi, 276003 Shandong China; 3 Jinzhou Medical University, Jinzhou, Liaoning Province China

**Keywords:** Osteonecrosis of the femoral head, MMP-9, TIMP-1, Bone remodelling

## Abstract

**Background:**

Matrix metalloproteinase-9 (MMP-9) and tissue inhibitor of metalloproteinases-1 (TIMP-1) are involved in the pathological mechanism of osteonecrosis of the femoral head (ONFH). This study aimed to investigate the relationship of serum MMP-9, TIMP-1, and the MMP-9/TIMP-1 ratio with disease severity in patients with nontraumatic ONFH.

**Methods:**

Serum levels of MMP-9 and TIMP-1 among 102 nontraumatic ONFH patients and 96 healthy individuals were determined by enzyme-linked immunosorbent assay (ELISA). Imaging severity was determined using the FICAT classification system. The Harris hip score (HHS) and visual analogue scale (VAS) were used to evaluate clinical progress. The correlations of serum MMP-9 and TIMP-1 levels with imaging severity and clinical progress was evaluated statistically. The diagnostic value of MMP-9 for NONFH disease severity was evaluated by examining receiver operating characteristic (ROC) curves.

**Results:**

The serum MMP-9 levels and the MMP-9/TIMP-1 ratio were significantly increased in patients with ONFH compared to normal controls, and TIMP-1 levels did not differ between the two groups. Serum MMP-9 levels and the MMP-9/TIMP-1 ratio were positively correlated with FICAT stage and VAS and were negatively correlated with the HHS score. The ROC curve results indicated that MMP-9 could be used as a potential marker of nontraumatic ONFH imaging progression.

**Conclusions:**

We hypothesize that increased MMP-9 expression and an imbalance in the MMP-9/TIMP-1 ratio play a role in the development of ONFH and are correlate with the severity of ONFH. The determination of MMP-9 can be a useful tool to assess the severity of the disease in patients with nontraumatic ONFH.

## Background

Nontraumatic osteonecrosis of the femoral head (ONFH) is a common progressive disease of the hip in orthopaedic clinics [1]. There is ongoing debate regarding the pathogenesis of ONFH, and approximately 80% of cases are associated with high-dose cortisol therapy and alcohol abuse, which may lead to intraosseous hypertension, disturbances in bone remodelling, intravascular coagulation, and osteocyte apoptosis that may contribute to the pathogenesis of ONFH [2]. Moreover, the underlying cause of osteonecrosis is the disruption of blood flow, leading to osteonecrosis [3].

Epidemiological surveys suggest that more than 20 million people worldwide may have ONFH, and most patients are young, with an average age of only 38 years [4]. Early diagnosis of ONFH is difficult; furthermore, if untreated, progressive ONFH can lead to collapse of the femoral head and secondary osteoarthritis requiring total hip arthroplasty (THA) [5]. However, many young patients face the risk of postoperative complications as well as revision after THA. Several treatment modalities have been developed to slow its progression with some success, such as core decompression, femoral osteotomy, and vascularized bone grafting [6]. Recent evidence suggests that vascularized iliac grafting is suitable for small- to medium-sized lesions in young patients and can relieve pain and avoid THA [7].

Matrix metalloproteinases (MMPs) are a family of zinc-dependent protein hydrolases capable of degrading almost all components of the extracellular matrix (ECM) and are regulated by tissue inhibitors of metalloproteinases (TIMPs) [8]. In addition to their primary role in maintaining the dynamic homeostasis of the ECM, these molecules can play a broader function in tissue remodelling, angiogenesis, regulation of the apoptotic process, and alteration of cell adhesion and migration [9]. Under normal physiological conditions, the expression levels of MMPs are extremely low, whereas under pathological conditions, such as inflammatory factors, growth factors, and oxidative stress, MMP expression levels are significantly upregulated.

Matrix metalloproteinase-9 (MMP-9) is widely expressed in osteoblasts and osteoclasts and is thought to be the main endopeptidase that destages the bone matrix during bone resorption, and metalloproteinase inhibitor-1 (TIMP-1) may bind to MMP-9 at an approximately 1:1 ratio to inhibit its enzymatic activity [10, 11]. Previous studies have suggested that MMP-9 is involved in the pathogenesis of ONFH [12]. In addition, MMP-9 has been reported as a potential biomarker for osteoarthritis and as a biomarker of bone remodelling and healing after arthroscopic acromioplasty [13, 14]. However, no previous study has investigated the relationship between serum MMP-9 and TIMP-1 levels and ONFH. Therefore, based on these findings, the purpose of this investigation is to explore the relationship between serum MMP-9 and TIMP-1 levels and the severity of ONFH, as well as their potential use as biomarkers of bone repair status after vascularized iliac grafting.

## Methods

### Study subjects

All aspects of the study were approved in advance by the Institutional Review Board of Linyi People’s Hospital, and informed written consent was obtained from each individual to participate in the study. A total of 199 people were included in this study consisting of 102 ONFH patients (36 men and 24 women) and 97 age- and sex-matched healthy controls (36 men and 24 women) who underwent physical examination during the same period. All ONFH patients and healthy individuals were recruited from Linyi City People's Hospital from July 2021 to July 2022. The diagnosis of ONFH is made by reference to symptoms, signs, and imaging of the hip. In this study, the aetiologic classification of ONFH was based on the aetiologic Classification Criteria of ARCO on Femoral Head Osteonecrosis [15, 16]: patients with steroid-induced ONFH had an intake history of a cumulative dose of glucocorticoids greater than 2 g prednisone or equivalent within 3 months and an interval between ONFH diagnosis and glucocorticoid discontinuation < 2 years. Patients with alcohol-induced ONFH had a history of high-dose continuous alcohol consumption (minimum dose of alcohol intake more than 400 ml/week and duration of alcohol exposure more than 6 months). The exclusion criteria for patients with ONFH were as follows: patients with a history of hip injury or fracture; patients with a history or evidence of metabolic bone disease, including bone tumours, malignancies with bone metastases, hyperparathyroidism or hypoparathyroidism, and Paget disease; patients who received hormones and other drugs that affect bone metabolism in the past 3 months; and patients with osteoarthritis or other large joint diseases. The inclusion criteria of the healthy control group were as follows: 1. age 25–60 years old; and 2. no tumour, cardiovascular disease or other underlying diseases were found. The exclusion criteria were the same as those for the ONFH group.

### Laboratory tests

Blood samples were obtained early in the morning after an overnight fast from all ONFH patients and healthy individuals on admission, as well as patients with vascularized iliac bone grafting at the first week, first month, second month, and third month after surgery. The serum was separated by centrifugation at 4 °C/3000 × g for 10 min and frozen at -80 °C until analysis. Serum MMP-9 and TIMP-1 levels were tested using an enzyme-linked immunosorbent assay (ELISA) kit (Cusabio, Wu Han, China), and the serum sample was diluted 100 times. The intralot variability was < 8%, and the interlot variability was < 10%. Finally, the lower limit of detection of this ELISA for MMP-9 and TIMP-1 was 0.312 ng/mL and 0.390 ng/mL, respectively. The lower limit of detection (LLD) was defined as the lowest protein concentration that could be differentiated from zero. The mean O.D. value of 20 replicates of the zero standard added by their three standard deviations. The experimental procedure was performed strictly according to the manufacturer's instructions, and all results were repeated twice and averaged.

### Radiological progression and assessment of symptom severity

Imaging progression was assessed according to the FICAT classification [17]: stage 1 is normal radiographic imaging with clinical signs and MRI changes; stage 2 includes sclerotic and cystic changes without femoral flattening or crescentic signs on radiographs; stage 3 includes crescentic signs with preserved joint space; and stage 4 is defined as femoral flattening and arthritic changes of the hip. The area of femoral head necrosis was measured according to the method described by Steinberg [18]. Imaging results were read and evaluated by an experienced radiologist who was blinded to the patient.

The severity of ONFH was assessed using the VAS score [19] and the HHS score [20].

### Post hoc statistical power calculation

Statistical power (1-β) was calculated using the Energy and Sample Size Calculator (http://powerandsamplesize.com) based on data with different mean MMP-9/TIMP-1 ratios per group, standard deviations, and the number of patients registered per group [21]. Statistical power was considered strong when > 0.8. The calculation was performed as follows.

$$\begin{array}{l}1-\beta=\phi\left(z-z_{1-\alpha/2}\right)+\phi\left(-z-z_{1-\alpha/2}\right)\\z=\left(\mu_A-\mu_B\right)/\sigma\left(\frac1{n_A}+\frac1{n_B}\right)\end{array}\\$$

### Statistics

Statistical analysis was performed using SPSS 26.0 software, and all data are expressed as the mean ± standard deviation or median. Demographic and clinical data between ONFH patients and healthy individuals were analysed using chi-square tests or independent samples t tests, and one-way analysis of variance was used for comparisons between patient subgroups, followed by the Tukey or Tamhane post hoc tests. The correlations of the serum levels of MMP-9 and TIMP-1 with the clinical severity and imaging progression of patients was determined by Spearman’s or Pearson’s correlation analysis. The area under the curve (AUC) of the MMP-9/TIMP-1 ratio was determined by subject operating characteristic (ROC) curves. *p* < 0.05 was considered a statistically significant difference.

## Results

### Demographic information

**Fig. 1:**
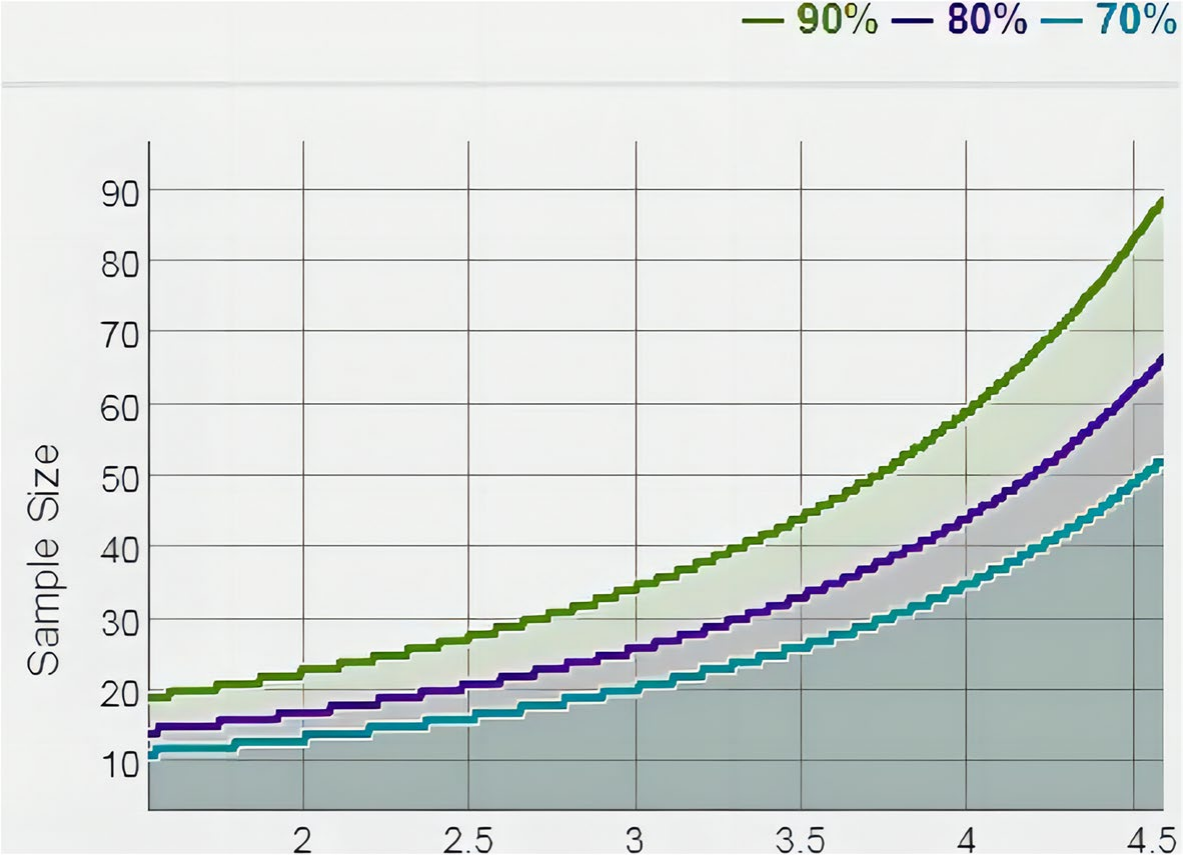
Statistical power determined by mean and sample size. Statistical power: green line for 0.9, purple line for 0.8, and blue line for 0.7

Demographic data are shown in Table 1. The 105 patients included 60 males and 42 females, with a mean age of 45.45 ± 10.15 years. There were 41 cases of alcoholic femoral head necrosis, 26 cases of hormonal femoral head necrosis, and 35 cases of idiopathic femoral head necrosis. There were 13 cases of FICAT stage 1, 24 cases of FICAT stage 2, 33 cases of FICAT stage 3, and 32 cases of FICAT stage 4. Of the 97 healthy subjects, 56 were male and 41 were female, with a mean age of 43.21 ± 9.49 years. age, the sex distribution of ONFH patients, and BMI were not significantly different from those of healthy controls. A statistical weight of ~ 1 was calculated, indicating that a sample size of 102 is sufficient to draw this conclusion (Fig. 1).

**Table 1:** Demographic data for ONFH patients and healthy controls

	ONFH patients (*n* = 102)	Healthy controls (*n* = 97)	*P* value
Age (Y)	45.45 ± 10.15	43.21 ± 9.49	0.109
Sex (F/M)	60/42	56/41	0.876
BMI (kg/m^2^)	24.15 ± 3.38	23.79 ± 2.80	0.411
Disease duration (M)	29(3–136)	/	
VAS scores	4.79 ± 2.08	/	
HHS scores	61.93 ± 16.77	/	
FICAT stage (1/2/3/4)	13/24/33/32	/	
alcohol/Steroid/idiopathic (n)	41/26/35	/	
MMP-9 level (ng/ml)	1073.26 ± 564.34	587.84 ± 295.70	< 0.05
TIMP-1level (ng/ml)	186.52 ± 71.87	207.81 ± 85.34	0.058
MMP-9/TIMP ratio	7.17 ± 6.71	3.07 ± 1.48	< 0.05

All data are given as the mean value ± SD or median

### Serum MMP-9 and TIMP-1 expression in patients with femoral head necrosis

**Fig. 2:**
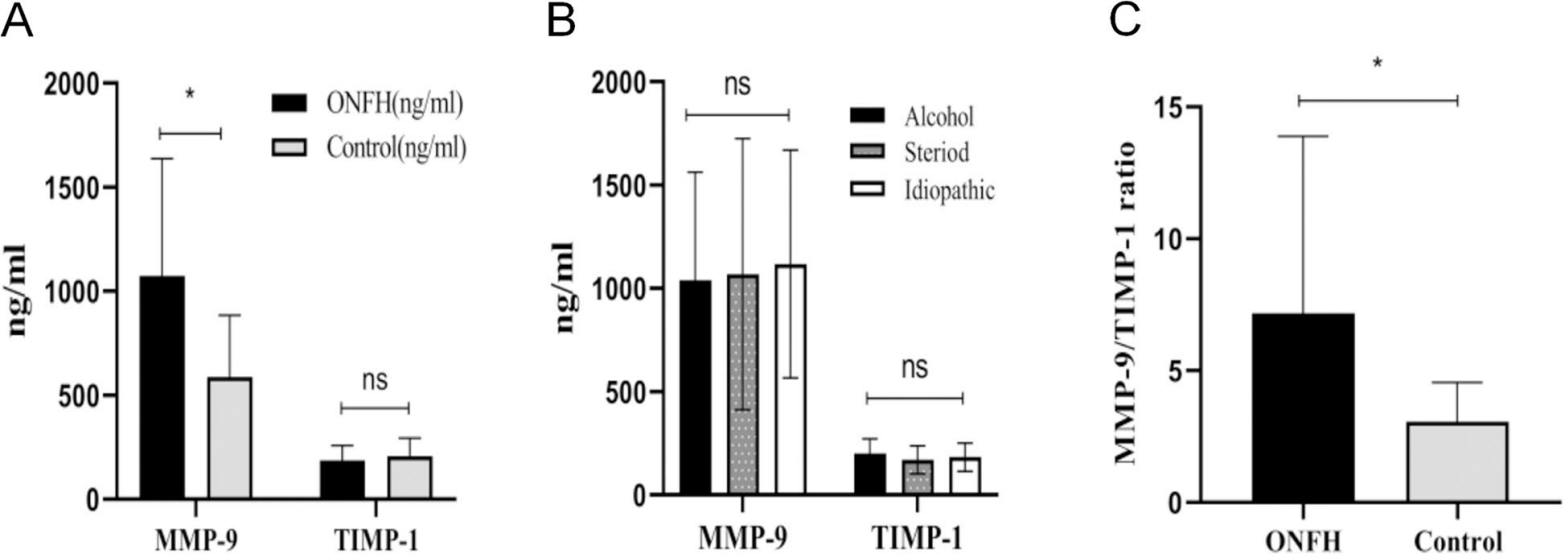
**A** Comparison of serum MMP-9 and TIMP-1 levels between ONFH patients and healthy controls. **B** Comparison of serum APN levels among different steroid use, alcohol use, and idiopathic groups. **C** Comparison of the MMP-9/TIMP-1 ratio between ONFH patients and healthy controls. Data are expressed as the mean ± SD. NS, *p* > 0.05; *p < 0.05

The expression of MMP-9 and TIMP-1 in serum was detected by enzyme-linked immunosorbent assay. Serum MMP-9 levels were significantly higher in ONFH patients than in healthy individuals (1073.26 ± 564.34 vs. 587.84 ± 295.70, *P* < 0.05; Fig. 2A), and serum TIMP-1 levels were lower in the necrotic group than in the healthy group, but the difference was not statistically significant (186.52 ± 71.87 vs. 207.81 ± 85.34, *P* = 0.058; Fig. 2A). There was no statistically significant difference in MMP-9 and TIMP-1 levels between aetiological groups (MMP9: alcohol group 1039.29 ± 522.79 ng/ml vs. steroid group 1067.82 ± 656.55 ng/ml vs. idiopathic group 1117.10 ± 551.53 ng/ml, F = 0.178, *P* = 0.837; TIMP1: alcohol group 200.26 ± 71.87 ng/ml vs. hormone group 169.62 ± 67.30 ng/ml vs. idiopathic 182.99 ± 68.11 ng/ml, F = 1.525, *P* = 0.223; Fig. 2B). In addition, we found that the MMP-9/TIMP-1 ratio and serum MMP-9/TIMP-1 ratio were significantly higher in ONFH patients than in healthy individuals (7.17 ± 6.71 vs. 3.07 ± 1.48, *P* < 0.05; Fig. 2C).

### Relationship between serum MMP-9 and its ratio to TIMP-1 and radiological progression and clinical severity of patients with femoral head necrosis

**Fig. 3:**
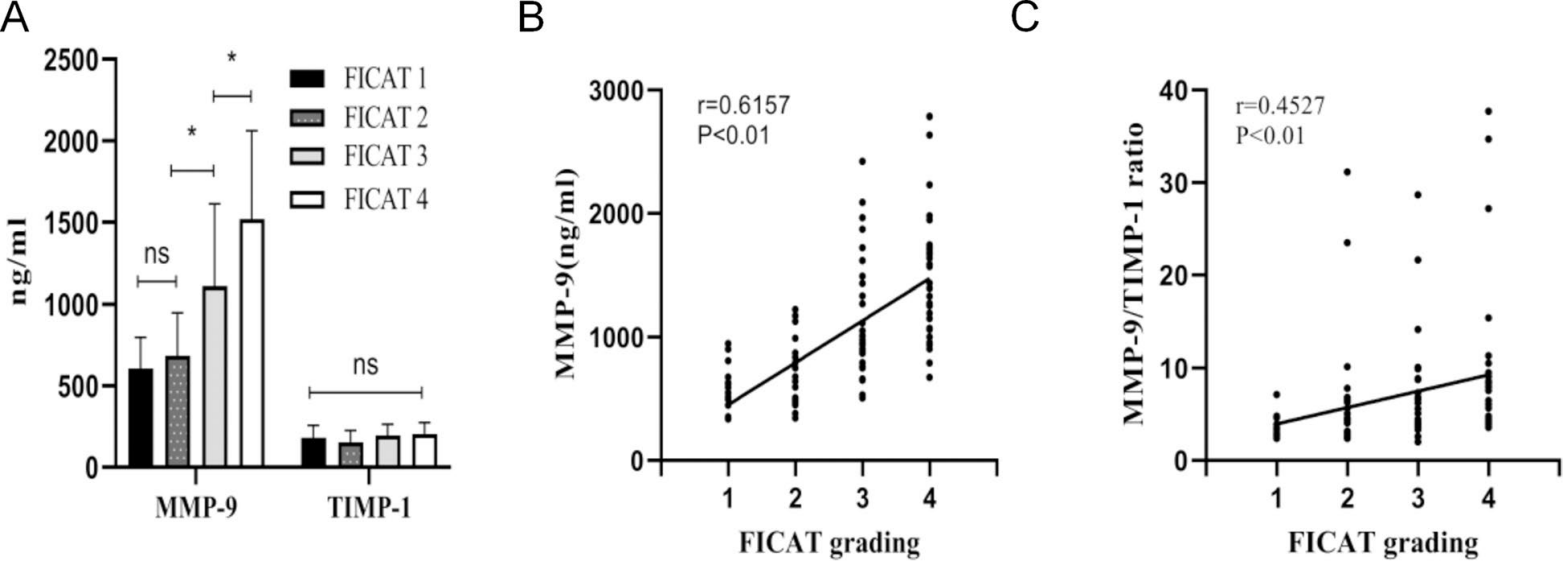
**A** Comparison of serum MMP-9 and TIMP-1 levels among different FICAT stages. **B** Correlation of serum MMP-9 levels with FICAT grade. **C** Correlation of MMP-9/TIMP-1 ratio with FICAT grade. NS, *p* > 0.05; *p < 0.05

Serum MMP-9 and TIMP-1 concentrations in 102 nontraumatic ONFH patients with different FICAT classifications are shown in Fig. 3A. Compared with FICAT stage 3 patients, serum MMP-9 levels were significantly higher in FICAT stage 4 nontraumatic ONFH patients (1108.78 ± 503.87 ng/ml vs. 1518.71 ± 542.83 ng/ml, *P* < 0.05), while MMP-9 levels were significantly higher in FICAT stage 3 patients than in FICAT stage 2 patients (683.48 ± 263.59 ng/ml vs. 1108.78 ± 503.87 ng/ml, *P* < 0.05), but there was no difference in serum MMP-9 levels between FICAT class I and FICAT class II patients (FICAT 1 606.21 ± 190.23 ng/ml vs. FICAT 2 683.48 ± 263.59 ng/ml, *P* = 0.958). Serum MMP-9 levels increased with the progression of FICAT staging and were positively correlated with FICAT staging (*r* = 0.6157, *P* < 0.05; Fig. 3B). TIMP-1 concentrations did not differ significantly between groups (FICAT 1 180.73 ± 75.70 ng/ml vs. F ICAT 2 153.70 ± 71.20 ng/ml vs. FICAT 3 196.17 ± 66.20 ng/ml vs. FICAT 4 203.55 ± 71.28 ng/ml, *p* = 0.056). We then analysed the MMP-9/TIMP-1 ratio, which gradually increased with the progression of FICAT grading (FICAT 1 3.68 ± 1.27 vs. FICAT 2 6.38 ± 6.83 ng/ml vs. FICAT 3 6.84 ± 5.54 ng/ml vs. FICAT 4 9.54 ± 8.26 ng/ml, *P* < 0.05) and was positively correlated with FICAT classification (*r* = 0.4527, *P* < 0.05; Fig. 3C).

**Fig. 4:**
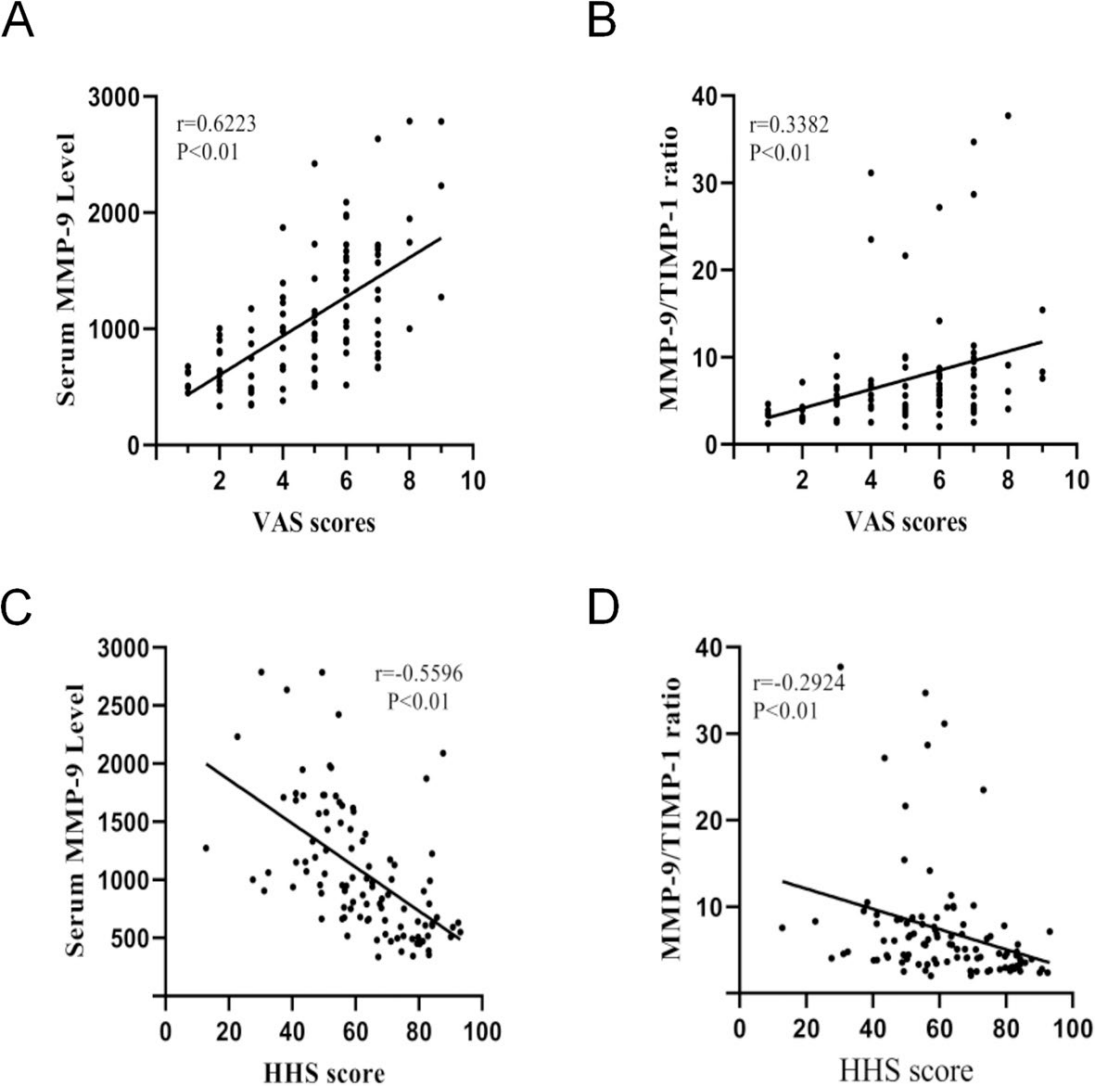
**A** Correlation of serum MMP-9 levels with VAS scores. **B** Correlation of the MMP-9/TIMP-1 ratio with VAS scores. **C** Correlation of serum MMP-9 levels with HHS scores. **D** Correlation of the MMP-9/TIMP-1 ratio with HHS scores

We further explored the relationship between serum MMP-9 levels and clinical severity as determined by the VAS score; the HSS score, serum MMP-9 levels, and MMP-9/TIMP-1 ratio were positively correlated with visual analogue score (VAS) (MMP-9: *r* = 0.6223, *P* < 0.05, Fig. 4A; MMP-9/TIMP-1: *r* = 0.3382, *P* < 0.05, Fig. 4C) and negatively correlated with HHS scores (MMP-9: *r* = -0.5596, *P* < 0.05, Fig. 4B; MMP-9/TIMP-1: *r* = -0.2924, *P* < 0.05, Fig. 4D). The results showed that MMP-9 levels were more strongly correlated with the clinical and radiographic findings of nontraumatic ONFH than the MMP-9/TIMP-1 ratio.

### ROC curve

**Fig. 5:**
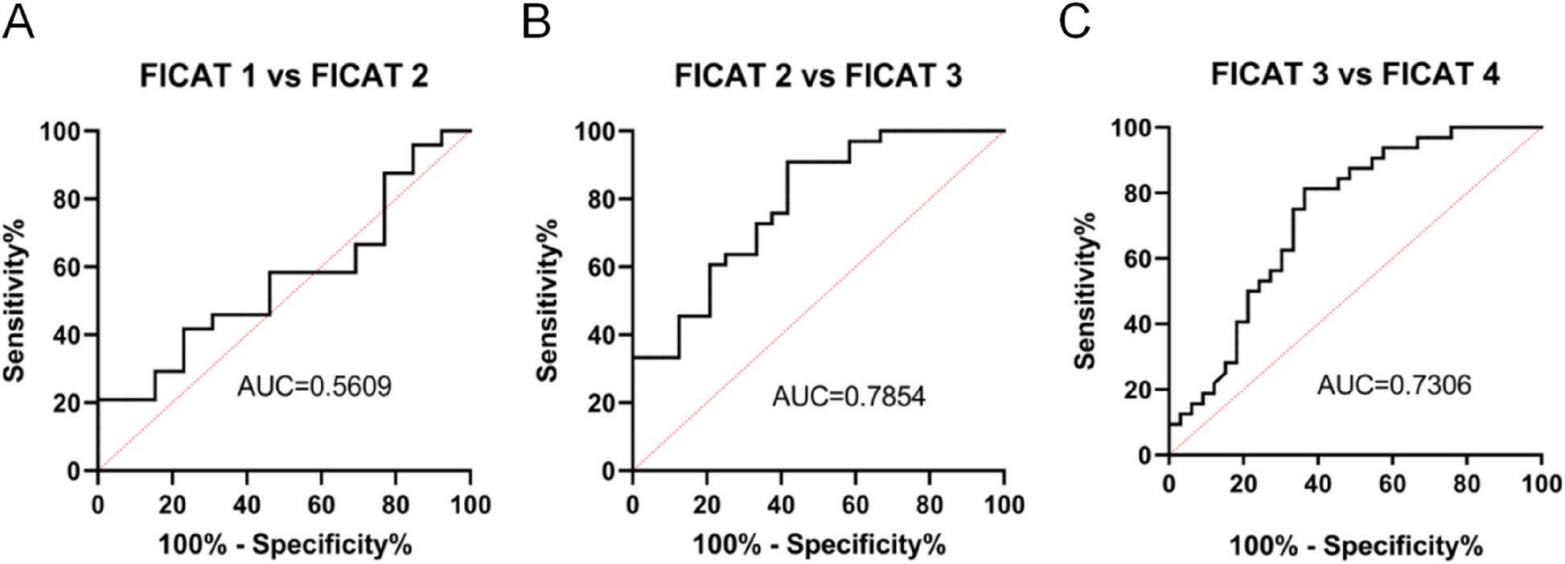
**A** ROC curve analysis of serum MMP-9 levels with regard to FICAT 1 vs. FICAT 2. **B** ROC curve analysis of serum MMP-9 levels with regard to FICAT 2 vs. FICAT 3. **C** ROC curve analysis of serum MMP-9 levels with regard to FICAT 3 vs. FICAT 4

We further used ROC curve analysis to explore the diagnostic value of MMP-9 in the severity of patients with ONFH. As shown in Fig. 5, the AUC of FICAT level 1 vs. level 2 was 0.561; FICAT level 2 vs. 3 had an AUC of 0.785, and FICAT level 3 vs. 4 had an AUC of 0.731. These findings suggest that elevated MMP-9 may serve as a potential marker of nontraumatic ONFH imaging progression.

## Discussion

In our study, serum MMP-9 levels were significantly elevated in patients with nontraumatic ONFH compared to healthy individuals, with no differences among the different aetiologies. In bone tissue, inflammatory cytokines such as IL-1, IL-6, and TNF-α induce MMP-9 expression in osteoblasts, while in osteoclasts, MMP-9 expression is activated by the RANKL system, which induces MMP-9 synthesis [22]. A large volume of published studies have shown that inflammatory cytokines, especially IL-6, are significantly elevated in the local microenvironment of patients with ONFH, and similarly, some studies have confirmed higher than normal levels of RANK and RANKL mRNA expression in the lesioned area of the femoral head [23, 24]. In addition, the hypoxic environment also has an effect on MMP-9 expression, and clinical and animal experiments have confirmed increased expression of MMP-2 and MMP-9 in myocardial ischaemic tissues, especially MMP-9, which was enhanced approximately 20-fold compared to controls [25]. Masuhara K found that local and plasma MMP-3 and MMP-9 levels were significantly elevated in patients with rapidly destructive hip osteoarthritis, and he attributed the elevated blood levels to a large amount of MMP-9 produced in the synovial tissue of the hip [26]. Therefore, we suggest that increased inflammatory cytokine and RANKL expression at the site of femoral head necrosis promotes the biosynthesis and activation of MMP-9, which promotes a shift from bone reconstructive homeostasis to bone matrix degradation. It is known that using steroids and alcohol decreases bone mass and inhibits bone formation [27, 28]. Thus, the levels of MMP-9 and TIMP-1 in patients with different aetiologies were further analysed. We found that a high level of MMP-9 was significantly associated not only with steroid-induced or alcoholic ONFH but also with idiopathic ONFH, suggesting that a high level of MMP-9 might be a natural process in the onset or progression of nontraumatic ONFH.

TIMP-1 is the inhibitor with the highest affinity for MMP-9, and in bone tissue, it is mainly synthesized by osteoblasts whose expression is inhibited by RANKL [29, 30]. Additionally, it is involved in the regulation of cell growth, differentiation, and apoptosis through a pathway independent of matrix metalloproteinases (MMPs) [31]. TIMP knockout mice showed a significant reduction in bone trabeculae in long bones and a corresponding increase in metalloproteinase activity. TIMP deficiency reduced osteoblast matrix deposition and mineralization; conversely, osteoclasts were hyperactivated [32]. Balanced expression of MMPs and TIMPs is essential to maintain healthy tissue integrity by regulating ECM catabolism. Overexpression of MMPs and abnormal ECM catabolism leading to an imbalance in bone remodelling is a major cause of many skeletal pathological states. In COPD patients with osteoporosis, circulating MMP-9 levels were increased, whereas TIMP-1 and -2 were not different and associated with bone loss (reduced bone mass and osteoporosis) [33]. In a further study, Zhang PF found that serum MMP-9/TIMP-1 ratios in COPD patients with osteoporosis were significantly higher (high MMP-9, low TIMP-1) than in patients with normal bone mineral density (BMD) and that these ratios were negatively correlated with BMD [34]. Grässel S found that the protein content of TIMP-1 in bone biopsy tissues of ONFH patients was significantly lower than that of MMP-9 [23]. In our study, in contrast, serum TIMP-1 concentrations did not change in ONFH patients, the circulating MMP-9/TIMP-1 ratio was significantly higher than that of healthy individuals, and the MMP-9/TIMP-1 ratio was positively correlated with FICAT stage. The imbalance of the MMP-9/TIMP-1 ratio may accelerate bone matrix degradation, resulting in osteoporosis under cartilage, the inability of bone tissue to withstand normal pressure load, and the destruction of microvessels caused by microfracture of bone trabeculae, which further leads to necrosis progression. Therefore, our results suggest that an imbalance in the MMP-9/TIMP-1 ratio may be one of the pathological mechanisms of ONFH.

This study was also the first to focus on the relationship between serum MMP-9 levels and its ratio to TIMP-1 with VAS scores and HSS scores. Serum MMP-9 levels and their ratio to TIMP-1 were positively correlated with pain levels as determined by VAS scores and negatively correlated with functional impairment as determined by HSS scores. Previous studies have demonstrated that MMP-9 plays an important role in inflammatory and painful processes, and the Escolano-Lozano F study found that elevated serum MMP-9 levels correlated with the severity of complex regional pain syndrome [35], and several other studies found that MMP-9 dysregulation was associated with acute migraine [36] and lumbar spinal stenosis pain [37]. Compared with the ratio of MMP-9/TIMP-1, the level of MMP-9 was better correlated with the severity of nontraumatic ONFH disease and could better reflect the disease status. Further ROC curve analysis showed that the elevation of MMP-9 was of great value in the evaluation of nontraumatic ONFH after FICAT-1 and may be a potential marker of imaging severity of nontraumatic ONFH.

This study has several limitations. First, this is a single-centre study with a relatively small sample, but the sample size has strong statistical power. Further studies on larger samples are needed. Second, only MMP-9 and TIMP-1 were examined in this study, and other MMPs were not investigated. Third, this study only evaluated the value of MMP-9 in the assessment of nontraumatic ONFH, and further studies are needed on its potential application in the assessment of therapeutic effects and other aspects.

## Conclusion

In conclusion, we hypothesize that increased MMP-9 expression and an imbalance in the MMP-9/TIMP-1 ratio play a role in the development of ONFH and are correlated with the severity of ONFH. Moreover, MMP-9 can be used as a useful tool to assess disease severity in patients with ONFH.

## Data Availability

Relevant data are available from the corresponding author upon reasonable request.

## Abbreviations

MMPs: Matrix metalloproteinase
TIMPs: Tissue inhibitor of metalloproteinases
ONFH: Osteonecrosis of the femoral head
ECM: Extracellular matrix
BMD: Bone mineral density

## References

[1] Shen X, Luo J, Tang X, et al. Deep learning approach for diagnosing early osteonecrosis of the femoral head based on magnetic resonance imaging [published online ahead of print, 2022 Oct 12]. J Arthroplasty. 2022: S0883-5403(22)00900-7. 10.1016/j.arth.2022.10.003. 10.1016/j.arth.2022.10.003 36243276

[2] Kubo T, Ueshima K, Saito M, Ishida M, Arai Y, Fujiwara H (2016). Clinical and basic research on steroid-induced osteonecrosis of the femoral head in Japan. J Orthop Sci.

[3] Cui Q, Jo WL, Koo KH (2021). ARCO Consensus on the Pathogenesis of Non-traumatic Osteonecrosis of the Femoral Head. J Korean Med Sci.

[4] Cui L, Zhuang Q, Lin J (2016). Multicentric epidemiologic study on six thousand three hundred and ninety five cases of femoral head osteonecrosis in China. Int Orthop.

[5] Mont MA, Zywiel MG, Marker DR, McGrath MS, Delanois RE (2010). The natural history of untreated asymptomatic osteonecrosis of the femoral head: a systematic literature review. J Bone Joint Surg Am.

[6] Chughtai M, Piuzzi NS, Khlopas A, Jones LC, Goodman SB, Mont MA (2017). An evidence-based guide to the treatment of osteonecrosis of the femoral head. Bone Joint J.

[7] Seyler TM, Marker DR, Ulrich SD, Fatscher T, Mont MA (2008). Nonvascularized bone grafting defers joint arthroplasty in hip osteonecrosis. Clin Orthop Relat Res.

[8] Hardy E, Fernandez-Patron C (2020). Destroy to rebuild: the connection between bone tissue remodeling and matrix metalloproteinases. Front Physiol.

[9] Cui N, Hu M, Khalil RA (2017). Biochemical and biological attributes of matrix metalloproteinases. Prog Mol Biol Transl Sci.

[10] Kou L, Jiang X, Lin X (2021). Matrix Metalloproteinase Inspired Therapeutic Strategies for Bone Diseases. Curr Pharm Biotechnol.

[11] Baker AH, Edwards DR, Murphy G (2002). Metalloproteinase inhibitors: biological actions and therapeutic opportunities. J Cell Sci.

[12] Fang S, Li Y, Chen P (2018). Osteogenic effect of bone marrow mesenchymal stem cell-derived exosomes on steroid-induced osteonecrosis of the femoral head. Drug Des Devel Ther.

[13] Li S, Wang H, Zhang Y (2021). COL3A1 and MMP9 serve as potential diagnostic biomarkers of osteoarthritis and are associated with immune cell infiltration. Front Genet.

[14] Galliera E, Randelli P, Dogliotti G (2010). Matrix metalloproteases MMP-2 and MMP-9: are they early biomarkers of bone remodelling and healing after arthroscopic acromioplasty?. Injury.

[15] Yoon BH, Jones LC, Chen CH (2019). Etiologic classification criteria of ARCO on femoral head osteonecrosis part 2: alcohol-associated osteonecrosis. J Arthroplasty.

[16] Yoon BH, Jones LC, Chen CH (2019). Etiologic classification criteria of ARCO on femoral head osteonecrosis part 1:glucocorticoid-associated osteonecrosis. J Arthroplasty.

[17] Cohen-Rosenblum A, Cui Q (2019). Osteonecrosis of the femoral head. Orthop Clin North Am.

[18] Steinberg ME, Hayken GD, Steinberg DR (1995). A quantitative system for staging avascular necrosis. J Bone Joint Surg Br.

[19] Reed MD, Van Nostran W (2014). Assessing pain intensity with the visual analog scale: a plea for uniformity. J Clin Pharmacol.

[20] Shi HY, Mau LW, Chang JK, Wang JW, Chiu HC (2009). Responsiveness of the Harris Hip Score and the SF-36: five years after total hip arthroplasty. Qual Life Res.

[21] Chow S, Shao J, Wang H (2008). Sample Size Calculations in Clinical Research.

[22] Kusano K, Miyaura C, Inada M (1998). Regulation of matrix metalloproteinases (MMP-2, -3, -9, and -13) by interleukin-1 and interleukin-6 in mouse calvaria: association of MMP induction with bone resorption. Endocrinology.

[23] Grässel S, Beckmann J, Rath B (2010). Expression profile of matrix metalloproteinase-2 and -9 and their endogenous tissue inhibitors in osteonecrotic femoral heads. Int J Mol Med.

[24] Samara S, Dailiana Z, Chassanidis C (2014). Expression profile of osteoprotegerin, RANK and RANKL genes in the femoral head of patients with avascular necrosis. Exp Mol Pathol.

[25] Ueland T, Yndestad A, Øie E (2005). Dysregulated osteoprotegerin/RANK ligand/RANK axis in clinical and experimental heart failure. Circulation.

[26] Masuhara K, Nakai T, Yamaguchi K, Yamasaki S, Sasaguri Y (2002). Significant increases in serum and plasma concentrations of matrix metalloproteinases 3 and 9 in patients with rapidly destructive osteoarthritis of the hip. Arthritis Rheum.

[27] Chotiyarnwong P, McCloskey EV (2020). Pathogenesis of glucocorticoid-induced osteoporosis and options for treatment. Nat Rev Endocrinol.

[28] Luo Z, Liu Y, Liu Y, Chen H, Shi S, Liu Y (2017). Cellular and molecular mechanisms of alcohol-induced osteopenia. Cell Mol Life Sci.

[29] Bord S, Horner A, Beeton CA, Hembry RM, Compston JE (1999). Tissue inhibitor of matrix metalloproteinase-1 (TIMP-1) distribution in normal and pathological human bone. Bone.

[30] Paiva KBS, Granjeiro JM (2017). Matrix metalloproteinases in bone resorption, remodeling, and repair. Prog Mol Biol Transl Sci.

[31] Egea V, Zahler S, Rieth N (2012). Tissue inhibitor of metalloproteinase-1 (TIMP-1) regulates mesenchymal stem cells through let-7f microRNA and Wnt/β-catenin signaling. Proc Natl Acad Sci U S A.

[32] Chen Y, Aiken A, Saw S, Weiss A, Fang H, Khokha R (2019). TIMP loss activates metalloproteinase-TNFα-DKK1 axis to compromise Wnt signaling and bone mass. J Bone Miner Res.

[33] Bolton CE, Stone MD, Edwards PH, Duckers JM, Evans WD, Shale DJ (2009). Circulating matrix metalloproteinase-9 and osteoporosis in patients with chronic obstructive pulmonary disease. Chron Respir Dis.

[34] Zhang PF, Pan L, Luo ZY, Zhao HJ, Cai SX (2013). Interrelationship of circulating matrix metalloproteinase-9, TNF-α, and OPG/RANK/RANKL systems in COPD patients with osteoporosis. COPD.

[35] Escolano-Lozano F, Gries E, Schlereth T (2021). Local and systemic expression pattern of MMP-2 and MMP-9 in complex regional pain syndrome. J Pain.

[36] Leira R, Sobrino T, Rodríguez-Yáñez M, Blanco M, Arias S, Castillo J (2007). Mmp-9 immunoreactivity in acute migraine. Headache.

[37] Lee J, Choi H, Park C, Jeon S, Yune T (2021). Jmjd3 mediates neuropathic pain by inducing macrophage infiltration and activation in lumbar spinal stenosis animal model. Int J Mol Sci.

